# Cohort study ON Neuroimaging, Etiology and Cognitive consequences of Transient neurological attacks (CONNECT): study rationale and protocol

**DOI:** 10.1186/s12883-015-0295-3

**Published:** 2015-03-15

**Authors:** Frank G van Rooij, Anil M Tuladhar, Roy PC Kessels, Sarah E Vermeer, Bozena M Góraj, Peter J Koudstaal, David G Norris, Frank-Erik de Leeuw, Ewoud J van Dijk

**Affiliations:** Department of Neurology, Donders Institute for Brain, Cognition and Behaviour, Centre for Neuroscience, Radboud University Medical Center, PO Box 9101, 6500 HB Nijmegen, Netherlands; Department of Medical Psychology, Radboud University Medical Center, PO Box 9101, 6500 HB Nijmegen, Netherlands; Department of Geriatrics, Radboud University Medical Center, PO Box 9101, 6500 HB Nijmegen, Netherlands; Donders Institute for Brain, Cognition and Behaviour, Centre for Cognition, Radboud University Nijmegen, 6500 HE Nijmegen, Netherlands; Department of Neurology, Rijnstate Hospital, PO Box 9555, 6800 TA Arnhem, Netherlands; Department of Radiology, Radboud University Medical Center, PO Box 9101, 6500 HB Nijmegen, Netherlands; Erasmus Medical Center, Department of Neurology, PO Box 2040, 3000 CA Rotterdam, Netherlands; Donders Institute for Brain, Cognition and Behaviour, Centre for Cognitive Neuroimaging, Radboud University Nijmegen, 6500 HE Nijmegen, Netherlands

**Keywords:** Transient ischemic attack, Transient neurological attack, Cognition, Prospective, Observational, Cohort

## Abstract

**Background:**

Transient ischemic attacks (TIA) are characterized by acute onset focal neurological symptoms and complete recovery within 24 hours. Attacks of nonfocal symptoms not fulfilling the criteria for TIA but lacking a clear alternative diagnosis are called transient neurological attacks (TNA). Although TIA symptoms are transient in nature, cognitive complaints may persist. In particular, attacks consisting of both focal and nonfocal symptoms (mixed TNA) have been found to be associated with an increased risk of dementia. We aim to study the prevalence, etiology and risk factors of cognitive impairment after TIA or TNA.

**Methods/Design:**

CONNECT is a prospective cohort study on cognitive function after TIA and TNA. In total, 150 patients aged ≥45 years with a recent (<7 days after onset) TIA or TNA and no history of stroke or dementia will be included. We will classify events as: TIA, nonfocal TNA, or mixed TNA. Known short lasting paroxysmal neurological disorders like migraine aura, seizures and Ménière disease are excluded from the diagnosis of TNA. Patients will complete a comprehensive neuropsychological assessment and undergo MRI <7 days after the qualifying event and again after six months. The primary clinical outcomes will be cognitive function at baseline and six months after the primary event. Imaging outcomes include the prevalence and evolution of DWI lesions, white matter hyperintensities and lacunes, as well as resting state networks functionality and white matter microstructural integrity. Differences between types of event and DWI, as well as determinants of both clinical and imaging outcomes, will be assessed.

**Discussion:**

CONNECT can provide insight in the prevalence, etiology and risk factors of cognitive impairment after TIA and TNA and thereby potentially identify a new group of patients at increased risk of cognitive impairment.

## Background

Transient ischemic attacks (TIAs) are characterized by acute onset focal neurological symptoms of vascular origin that resolve completely within 24 hours [1]. Often patients are encountered with a myriad of acute onset and short-lasting nonfocal symptoms, which do not fulfill the criteria for TIA and might have another cause than focal cerebral ischemia. In the absence of a clear alternative cause, these attacks are referred to as transient neurological attacks (TNAs) [2]. TNAs are almost as prevalent as TIAs, but their etiology is unknown [3].

In contrast to the definition of TIA, which states symptom resolution within 24 hours, many patients report cognitive problems afterwards [4]. In addition, particularly patients with a TNA or a mixture of focal and nonfocal symptoms (mixed TNA) have an increased risk of dementia [5].

Diffusion-weighted imaging (DWI) studies show signs of cytotoxic edema beyond the point of symptom resolution in 30% of TIA patients [6]. Cerebrovascular damage, even in the absence of clinical signs of stroke, may lead to cognitive decline [7-9]. Moreover, reduced microstructural integrity and functional connectivity are associated with worse cognitive performance [10,11]. The influence of DWI lesions and other cerebrovascular damage on the cognitive outcome of TNA patients is unknown, In addition, the cognitive profile of TIA and TNA patients is unclear.

Previous studies on cognitive function after TNA are scarce and have focused almost exclusively on TIA patients [12-14]. These were mostly cross-sectional studies, without brain imaging and information on previous cerebrovascular events. Furthermore, cognitive testing was often performed several months to years after the initial event. Since the risk of new stroke is highest shortly after TIA, a delay in testing can influence cognitive results [15].

Establishing a relationship between TIA or TNA and cognitive decline could identify a group of patients at risk of cognitive impairment and dementia and provides an opportunity to clarify how transient neurological symptoms relate to lasting cognitive consequences. By widening the scope beyond TIA to ‘TNAs’ with nonfocal and mixed symptoms, the previously described but not understood association between TNA and worse cognitive outcome can be prospectively investigated. Finally, performing brain imaging in these patients could provide insight in the early developmental mechanisms of dementia.

We therefore set up the Cohort study ON Neuroimaging, Etiology and Cognitive consequences of Transient neurological attacks (CONNECT); a prospective cohort study on cognitive function and its determinants in patients with a TIA or TNA and aged ≥45 years.

## Methods/Design

### Study design

CONNECT is a prospective cohort study on the cognitive function of patients with a TIA or TNA and aged ≥45 years. Patient recruitment takes place at a university hospital and a large regional nonacademic hospital. The Medical Review Ethics Committee region Arnhem-Nijmegen approved the study (NL31651.091.10) and written informed consent is obtained from all participants.

### Objectives

The primary objective of our study is to determine the course and profile of cognitive function and the causes of cognitive impairment after TIA and TNA, and its relationship with DWI abnormalities. Secondary objectives are to determine the prevalence and course of subjective cognitive complaints, depressive symptoms, fatigue and sleep disorders and their effect on cognitive outcome in patients with TIA or TNA.

### Study population

All consecutive patients referred to the specialized TIA clinics of the Radboud university medical center and the Rijnstate Hospital will be screened for eligibility. Patients with a final diagnosis of TIA or TNA within seven days after onset and aged ≥45 years at the time of the qualifying event are considered eligible for participation. TIA is defined as a sudden onset focal loss of brain function of vascular origin with complete resolution of focal symptoms within 24 hours [1]. TNA is defined as an attack of sudden nonfocal neurological symptoms that completely resolve within 24 hours, without clear evidence for migraine, epilepsy, Ménière disease, hyperventilation, cardiac syncope, hypoglycaemia, or orthostatic hypotension [5]. In order to minimize the influence of concomitant neurological disorders on cognitive function, the following exclusion criteria will be applied:

1. Prior stroke with clinical symptoms.
2. Prior diagnosis of dementia.
3. Intracerebral space-occupying lesion.
4. Prior neurological disease which can influence cognitive function (e.g. Parkinson’s disease, multiple sclerosis).
5. Inability to undergo MRI.

Prior TIA and TNA are not a reason for exclusion.

## Procedures

Eligible patients will be recruited at specialized TIA clinics. We intend to include 150 patients and expect to complete inclusion over a 3-year period. Patients formally enter the study after informed consent and written approval. Baseline assessments will take place during admission to the TIA clinic at the participating center and follow-up is performed six months later at the Radboud university medical center and Donders Institute for Brain, Cognition and Behaviour. Baseline and follow-up assessments are summarized in Table 1.

**Table 1:** **Schedule of assessments**

**Assessment**	**Baseline**	**Follow-up**
**Demographics**		
Age, sex	X	
Education	X	
Working status	X	
Marital status	X	
**Structured assessment of TIA/TNA presentation**		
Duration of symptoms	X	
Number of episodes in week before inclusion	X	
Focal symptoms*	X	
Nonfocal symptoms*	X	
Incident focal symptoms*		X
Incident nonfocal symptoms*		X
**Medical history**		
Hypertension	X	X
Dyslipidemia	X	X
Diabetes mellitus	X	X
Atrial fibrillation	X	X
Other cardiovascular diseases^†^	X	
First-degree relatives with cardiovascular disease	X	
Smoking status	X	X
Alcohol consumption	X	X
Drug abuse	X	X
Hypercoagulability	X	
Acute infection	X	
Migraine	X	
Previous TIA/TNA	X	
Carotid endarterectomy	X	X
Epilepsy	X	X
Depression	X	X
Current medication use	X	X
Incident cardiovascular events^†^		X
Incident TIA, cerebral infarction or hemorrhage		X
**Physical examination**		
Length and weight, BMI	X	
Blood pressure	X	
Neurological examination	X	
**ECG**		
(Paroxysmal) atrial fibrillation	X	
Ischemia	X	
Left ventricular hypertrophy	X	
**Fasting laboratory investigations**		
Glucose	X	
Lipid profile	X	
Creatinine	X	
**Neuropsychological assessment**		
*Global cognitive function*		
Mini Mental State Examination	X	
Frontal Assessment Battery	X	X
*Episodic memory*		
Rey Auditory Verbal Learning Test	X	X
*Executive function*		
Verbal fluency	X	X
Stroop Color Word Test (interference score)	X	X
Brixton Spatial Anticipation Test	X	X
*Information processing speed*		
Symbol-Digit Modalities Test	X	X
Stroop Color Word Test (Cards I and II)	X	X
*Attention*		
Verbal Series Attention Test	X	X
*Subjective cognitive failures*		
Cognitive failures Questionnaire	X	X
*Depressive symptoms*		
Hospital Anxiety and Depression Scale	X	X
*Sleep disorders*		
Scales for Outcomes in Parkinson’s Disease - Sleep	X	X
*Fatigue*		
Checklist on Individual Strength	X	X
*Prior cognitive performance*		
Informant Questionnaire on Cognitive Decline in the Elderly	X	X
**MRI**		
Anatomical sequences (T1, T2, FLAIR, T2*)	X	X
Diffusion-weighted imaging	X	
MR angiography	X	
Susceptibility-weighted imaging		X
Diffusion tensor imaging		X
Resting-state functional MRI		X

Empty cells indicate no assessment. BMI indicates body-mass index; FLAIR, fluid-attenuated inversion recovery; TNA, transient neurological attack; ToF, time-of-flight.

*Presence of 18 predefined symptoms, both focal and nonfocal (see also Table 2).

^†^Myocardial infarction, coronary artery bypass grafting, percutaneous transluminal coronary angioplasty, valvular heart disease and peripheral revascularization procedures.

### Sample size and power calculation

The sample size is based on the ability to detect a difference in average cognitive performance between the three patient groups, expressed as z-score, equal to 0.5 standard deviation. With a power of 80% and a type-I error rate of 0.1, we need 50 patients per group to identify this difference.

### Assessments - baseline

All patients referred to the TIA clinic are analyzed and treated according to a dedicated TIA protocol, which includes investigations as recommended in international guidelines [16].

#### Symptom presentation

We will obtain a detailed history of the signs and symptoms of the qualifying event. The patient’s own account of events is noted, after which a structured interview consisting of 18 questions about the presence of specific neurological symptoms (nine focal and nine nonfocal symptoms, Table 2) will be administered. These symptoms are derived from the latest classification of the National Institute of Neurological Disorders and Stroke and previous research on nonfocal TNAs [1,5]. The duration of symptoms and number of episodes in the week before presentation are also recorded.

**Table 2:** **Structured assessment of transient focal and nonfocal neurological symptoms**

**Focal**	**Nonfocal**
Hemiparesis	Decreased consciousness or unconsciousness
Hemihypesthesia	Confusion
Dysphasia	Amnesia
Dysarthria	Unsteadiness
Hemianopia	Nonrotatory dizziness
Transient monocular blindness	Positive visual phenomena
Hemiataxia	Paresthesias
Diplopia	Bilateral weakness of arms or legs
Vertigo	Unwell feelings

All symptoms should have a sudden onset, rapid clearance and last <24 hours.

#### Demographics and medical history

Standardized, structured questionnaires will be used to obtain information on demographics and level of education (classified using seven categories, in accordance with the Dutch educational system: 1 being less than primary school and 7 reflecting an academic degree) [17].

The presence of established and potential stroke risk factors as classified by the American Heart Association will be determined by standardized, structured questionnaires [18]. Established risk factors include age, sex, history of cardiovascular disease in first-degree relatives, hypertension, dyslipidemia, diabetes mellitus, smoking, atrial fibrillation and cardiovascular disease (myocardial infarction, coronary artery bypass grafting, percutaneous transluminal coronary angioplasty, valvular heart disease and peripheral revascularization procedures). Potential risk factors include alcohol consumption (excess consumption defined as an intake of >200 g of pure alcohol per week), drug abuse, hypercoagulability, acute infection, a history of migraine, and the use of oral contraceptives. In addition, all patients will be asked about a history of TIA, carotid endarterectomy, epilepsy, and depression. Current medication use is recorded and classified according to the Anatomical Therapeutic Chemical (ATC) classification system (World Health Organization, WHO Collaborating Centre for drug statistics and methodology, http://www.whocc.no/atcddd/).

#### Physical examination

All patients will undergo a neurological examination. Blood pressure will be measured in a supine position after five minutes of rest. The average of three measurements will be used for analysis. Hypertension is defined as a systolic blood pressure ≥135 mmHg and/or a diastolic blood pressure ≥85 mmHg and/or the use of blood pressure-lowering medication.

#### Neuropsychological assessment

Participants will undergo an extensive neuropsychological assessment covering the main cognitive domains. Neuropsychological tests are administered by a trained examiner in a quiet, well-lit room and under standard circumstances. The Mini-Mental State Examination and Frontal Assessment Battery are used as screening tools for global cognitive function and executive function, respectively [19,20]. The three-trial version of the Rey Auditory Verbal Learning Test (RAVLT) will be applied to assess verbal episodic memory. This test also includes delayed free-recall and recognition trials [21]. To evaluate executive function three tests will be used; a verbal fluency test (naming as many animals and professions within 60 seconds each; response generation), the interference score of the abbreviated Stroop Color Word Test (response inhibition), and the Brixton Spatial Anticipation Test (rule detection) [22,23]. Information processing speed will be tested with the Symbol-Digit Modalities Test and Cards I and II of the abbreviated Stroop Color Word Test [24,25]. Attention is measured with the Verbal Series Attention Test [26].

In addition to cognitive tests, patients will complete several self-report questionnaires. Subjective cognitive failures will be registered with the modified Cognitive Failures Questionnaire (CFQ) [27]. Both nighttime sleep and daytime sleepiness will be assessed with the Scales for Outcomes in Parkinson’s Disease - Sleep, and the presence of depressive symptoms with the Hospital Anxiety and Depression Scale [28,29]. Furthermore, patients will fill out the Checklist on Individual Strength, a validated questionnaire on fatigue [30]. Finally, cognitive function prior to the qualifying event will be assessed by asking relatives to complete the Informant Questionnaire on Cognitive Decline in the Elderly (IQCODE) [31].

#### Ancillary investigations

##### MRI protocol

All included patients will undergo MRI of the brain as well as MR angiography of the carotid and vertebral arteries. MRI scanning will be performed on a 1.5-Tesla Magnetom Scanner (Siemens, Erlangen, Germany). The scanning protocol of the brain includes transversal T1-weighted spin echo sequence (TR/TE 532/10 ms; flip angle 90°; voxel size 0.8 × 0.6 × 5.0 mm); transversal fluid-attenuated inversion recovery (FLAIR) pulse sequence (TR/TE/T1 9000/108/2500 ms; voxel size 0.9 × 0.7 × 5.0 mm), transversal T2-weighted turbo spin echo sequence (TR/TE 5010/98 ms; voxel size 0.6 × 0.4 × 5.0 mm), transversal T2*-weighted gradient echo sequence (TR/TE 727/19.1 ms; voxel size 1.0 × 0.7 × 5.0 mm), and single-shot echo planar diffusion-weighted sequence (TR/TE 6300/134 ms; EPI factor 192; voxel size 1.2 × 1.2 × 5.0 mm) with diffusion weighting applied in three directions and a b-value of 1000 s/mm^2^. The scanning protocol of the cerebropetal vessels includes three-dimensional time-of-flight (ToF), phase contrast angiography (PCA), transversal T1-, T2- and proton density weighted sequences. Furthermore, a maximum intensity projection ToF and PCA will be reconstructed. The complete scanning protocol takes approximately 30 minutes. All cerebropetal vessel studies will be assessed for the presence and degree of stenosis by an experienced neuroradiologist.

##### Electrocardiogram

A conventional 12-lead electrocardiogram (ECG) will be performed and assessed by an experienced physician. The presence of atrial fibrillation, prior myocardial infarction and left ventricular hypertrophy is noted for every patient. In case of doubt a cardiologist is consulted for expertise.

##### Vena puncture

Fasting blood samples will be taken and analysis will include glucose, lipid profile and creatinine.

#### Classification of TNA

Three experienced stroke neurologists will independently adjudicate qualifying events as TIA, nonfocal TNA, or mixed TNA, based on the complete description of signs and symptoms as provided by the patient, the presence or absence of specific focal and nonfocal neurological symptoms, and the medical history. Fifty case descriptions will be assigned to each possible pair of classifying neurologists, such that each event is independently adjudicated by two specialists. In case of disagreement a consensus meeting including the third stroke neurologist will be held. The classifying neurologists will be blinded to the results of neuropsychological tests, brain imaging studies, or any other ancillary investigations. An attack containing at least one focal neurological symptom will be classified as TIA or mixed TNA, depending on the presence of additional nonfocal symptoms. When only nonfocal symptoms are present, the event is categorized as nonfocal TNA.

### Assessments - follow-up

Six months after baseline all patients are contacted for follow-up. In case of inability or unwillingness to undergo (part of) the follow-up investigations, the reasons are recorded. Follow-up measurements are summarized in Table 1.

#### Incident events

Patients will be asked whether they have experienced any of the 18 predefined neurological symptoms between baseline and follow-up (Table 2). Symptoms had to develop within seconds to minutes, last less than 24 hours and disappear completely. Reported symptoms are evaluated for these features by an investigator.

In addition, a standardized, structured questionnaire will be administered to determine the occurrence of new cardiovascular events between baseline and follow-up. Whenever an event is suspected, additional information on the exact date of diagnosis, treating physician and initiated therapies will be gathered. When necessary, the treating physician will be contacted for further information. All suspected events will be evaluated and adjudicated by a medical specialist trained in the specific field. The following items are assessed: TIA, ischemic and hemorrhagic stroke, myocardial infarction, coronary artery bypass graft, percutaneous transluminal coronary angioplasty, and carotid endarterectomy.

#### Neuropsychological assessment

A comprehensive neuropsychological assessment covering the main cognitive domains will be performed under the same conditions as baseline. To minimize material-specific learning effects, parallel versions are used for the verbal fluency task (items of clothing and fruit) and the RAVLT.

Subjective cognitive failures occurring between baseline and follow-up will be assessed with the modified CFQ. The presence of depressive symptoms, fatigue and sleep disturbances will be determined with the same questionnaires as at baseline [27-30]. Patients are specifically asked to reflect on the presence of these symptoms in the last two weeks. Furthermore, the influence of cognitive performance on daily functioning between baseline and follow-up as experienced by relatives will be determined by means of the IQCODE [31].

#### MRI protocol

MRI scanning at follow-up will be performed on a similar 1.5-Tesla Magnetom scanner (Siemens, Erlangen, Germany) as baseline. The scanning protocol consists of the following: whole brain 3-dimensional T1 magnetization-prepared rapid gradient-echo (MPRAGE) sequence (TR/TE/T1 2730/2.95/1000 ms; flip angle 7°; voxel size 1.0 × 1.0 × 1.0 mm), transversal FLAIR pulse sequence (TR/TE/T1 12220/85/2200 ms; voxel size 1.2 × 1.0 × 3.0 mm; slice gap 0.6 mm), transversal T2-weighted turbo spin echo sequence (TR/TE 7440/96 ms; voxel size 0.9 × 0.9 × 3.0 mm), transversal T2*-weighted gradient echo sequence (TR/TE 727/19.1 ms; voxel size 1.0 × 0.7 × 5.0 mm), gradient echo susceptibility weighted imaging sequence (TR/TE 49/40 ms; voxel size 0.8 × 0.7 × 1.0 mm), diffusion tensor imaging (DTI) (TR/TE 9100/98 ms; voxel size 2.2 × 2.2 × 2.2 mm; 7 unweighted scans, 61 diffusion weighted scans, with non co-linear orientation of the diffusion weighting gradient and b-value 1000 s/mm^3^), and resting state imaging using a gradient echo EPI (TR/TE 1870/35 ms; voxel size 3.5 × 3.5 × 3.0 mm; slice gap 0.5 mm). During resting state, patients will be told to relax with their eyes closed, without concentrating on anything particular. The complete scanning protocol takes approximately 60 minutes.

### Outcome measures

#### Clinical outcomes

The primary clinical outcome measure will be domain-specific cognitive performance at baseline and six months after the qualifying event. Raw cognitive test results at baseline and follow-up will be transformed into z-scores, based on the mean and standard deviation of the baseline tests. Next, domain-specific compound scores will be computed by averaging z-scores of tests assessing the same cognitive domain. The compound score for global cognitive function is the average of the z-scores of all tests [32]. Other clinical outcomes will be incident vascular events, subjective cognitive failures, fatigue, depressive symptoms, and sleep disorders.

#### Imaging outcomes

Two experienced raters will evaluate both baseline and follow-up brain MRI without knowledge of any clinical information.

##### Signs of acute brain infarction

A hyperintense lesion on DWI with a corresponding hypointensity on the apparent diffusion coefficient (ADC) map is considered cytotoxic edema and therefore a sign of acute brain infarction. In case of disagreement a consensus meeting will be held.

##### Lacunes of presumed vascular origin and territorial infarcts

A lacune is defined as a hypo-intensity on T1-weighted and FLAIR images >2 mm and ≤15 mm, ruling out enlarged perivascular spaces and infraputaminal pseudolacunes [33]. Territorial infarcts are defined as hypo-intense lesions on T1-weighted images >15 mm, with corresponding hyperintensity on FLAIR images [33]. After rating the presence of lacunes and territorial infarcts, clinical information regarding the qualifying, previous or incident TNAs, or any incident stroke, will be made available and the lesion will be categorized as silent or symptomatic, based on the clinical symptoms and lesion location. In case of disagreement a consensus meeting is held.

##### White matter hyperintensities of presumed vascular origin

White matter hyperintensities of presumed vascular origin (WMH) are defined as hyperintense lesions in the white matter on FLAIR, which are not or only slightly hypointense on T1-weighted sequences [33]. Gliosis surrounding infarcts is not considered WMH. Total WMH volume will be determined by an in-house developed, validated technique.

##### Brain volumetry

Probability maps for grey and white matter and cerebrospinal fluid will be computed using a six class segmentation tool in Statistical Parametric Mapping software (http://www.fil.ion.ucl.ac.uk/spm) (SPM 8) on the T1 images. Total volumes of grey and white matter are defined as the sum of all voxel volumes belonging to that tissue class. Total brain volume is taken as the sum of total grey and total white matter volume. After computation of co-registration parameters of the FLAIR image to the T1 image, these are used to bring both the FLAIR and WMH segmentation images into the patients anatomical reference frame. Resulting images will be visually checked for co-registration errors. Finally, WMH segmentations are resampled to and combined with the white matter probability maps, thus resulting in a WMH map (intersection of WMH and white matter) and normal-appearing white matter map (complement of WMH in white matter) in the T1 reference space.

##### Microbleeds

Microbleeds are defined as small, homogeneous, round or ovoid, hypo-intense foci on T2*-weighted images with a diameter of 2-10 mm [33]. Foci are not considered to be microbleeds when they are probable calcifications or iron deposits, symmetric hypo-intensities in the globus pallidus, flow void artifacts of pial blood vessels, or hypo-intense T2*-weighted signals inside an infarct, which are likely to be hemorrhagic transformation [34]. Microbleeds will be counted per hemisphere separately and stratified into lobar, deep and infratentorial lesions [34].

##### Diffusion tensor imaging

An in-house developed algorithm for patching artifacts from cardiac and head motion will be used for preprocessing of diffusion data [35]. The output of this Matlab (The Mathworks, Inc.) based program comprises the tensor derivates fractional anisotropy and mean diffusivity [36]. The mean unweighted image will be used to determine the coregistration parameters to the anatomical T1 image, which are then applied to all diffusion-weighted images and results [37]. All images will be visually checked for motion artifacts and coregistration errors.

##### Resting-state functional MRI

Resting-state images will be processed according to previously published methods, in order to gain motion corrected and spatially smoothed images with minimized effects of low and high non-neuronal noise [38], which in turn will be used to determine functional connectivity between different brain regions.

### Analysis

In the first analyses cognitive domain-specific composite z-scores are the outcome variables. Analysis of (co) variance will be performed to test for differences in cognitive performance at baseline and follow-up (using baseline mean and SD as reference) between TIA, mixed, and nonfocal TNA patients, adjusting for known determinants of cognitive function. The relationship between cognitive function and DWI lesions will be determined, adjusting for differences in other MRI parameters (WMH volume, total brain volume, silent brain infarcts, microbleeds). Analyses will be performed before and after exclusion of patients with incident stroke during follow-up. Next, multinominal logistic regression analysis will be used to identify determinants of cognitive decline among demographics, type of event, MRI parameters and vascular risk factors. Subsequently, the relationship between DTI parameters at follow-up and both explanatory determinants and cognitive function will be determined. Finally, functional connectivity will be compared between TIA, mixed, and nonfocal TNA and between strata of cognitive performance.

## Discussion

CONNECT provides an opportunity to identify patients who are not routinely included in cerebrovascular cohort studies but who are potentially at risk of cognitive decline [5]. The added value of our study to the current knowledge of cognition after TIA and TNA lies in the longitudinal design and the combination of comprehensive neuropsychological testing with both conventional and advanced neuroimaging. This could potentially provide more clarity in the causes and mechanisms of decline in cognitive function after short-lasting neurological deficits.

CONNECT may therefore contribute to the growing notion that TIA is not just a warning sign but has lasting consequences of its own, and identify TNA as a potential new group of patients at risk of cognitive decline.

## Abbreviations

ATC: Anatomical Therapeutic Chemical
CFQ: Cognitive Failures Questionnaire
DTI: Diffusion tensor imaging
DWI: Diffusion-weighted imaging
ECG: Electrocardiogram
FLAIR: Fluid-attenuated inversion recovery
IQCODE: Informant Questionnaire on Cognitive Decline in the Elderly
MIP: Maximum intensity projection
MPRAGE: Magnetization-prepared rapid gradient-echo
MRI: Magnetic resonance imaging
PCA: Phase contrast angiography
RAVLT: Rey Auditory Verbal Learning Test
SPM: Statistical Parametric Mapping
TIA: Transient ischemic attack
TNA: Transient neurological attack
ToF: Time-of-flight
WMH: White matter hyperintensities
